# Photophobia in neurologic disorders

**DOI:** 10.1186/s40035-017-0095-3

**Published:** 2017-09-20

**Authors:** Yiwen Wu, Mark Hallett

**Affiliations:** Human Motor Control Section, National Institute of Neurological Disorders and Stroke, National Institutes of Health, 10 Center Drive MSC 1428, Building 10, Room 7D37, Bethesda, MD 20892 USA

**Keywords:** Photophobia, Migraine, Blepharospasm, Progressive supranuclear palsy, Traumatic brain injury, Melanopsin

## Abstract

Photophobia is a common symptom seen in many neurologic disorders, however, its pathophysiology remains unclear. Even the term is ambiguous. In this paper, we review the epidemiology and clinical manifestations of photophobia in neurological disorders, including primary headache, blepharospasm, progressive supranuclear palsy, and traumatic brain injury, discuss the definition, etiology and pathogenesis, and summarize practical methods of diagnosis and treatment.

## Background

Photophobia refers to a sensory disturbance provoked by light. The term photophobia, derived from 2 Greek words, *photo* meaning “light” and *phobia* meaning “fear”, literally means “fear of light”. Patients may develop photophobia as a result of several different medical conditions, related to primary eye conditions, central nervous system (CNS) disorders and psychiatric disorders. Since the original literature described the manifestations of photophobia [1], many developments have taken place that makes it possible to establish a more precise picture of photophobia. In this paper, we will review its presence in neurological disorders, focusing on the definition, epidemiology, etiology, clinical manifestations, pathogenesis, diagnosis and treatment.

## Definition

In 1934, photophobia was first described and defined by Lebensohn as “exposure of the eye to light definitely induces or exacerbates pain” [1]. This definition emphasized that the core character is pain, but did not specify what kind of light (e.g., bright light or dim light) caused the symptom. In fact, photophobia is heterogeneous. Descriptions of photophobia vary among patients, and some of the heterogeneity comes from the different disorders that manifest this symptom. For example, some patients with migraine will deny that pain is a part of the experience at all but just prefer to be in a darkened room [2]. Thus, some researchers believe that photophobia involves not only the pain pathway, but also a limbic system pathway that superimposes an emotional discomfort leading to avoidance of light [3, 4]. Due to the evidence from basic research and the limitations of the earlier definition, Fine and Digre used the term “photo-oculodynia” to describe the pain or discomfort in the eye arising from a light source that should not be painful or discomforting under ordinary circumstances [5]. The term “photophobia” is defined by Digre and Brennan as a sensory state in which light causes discomfort in the eye or head; it may also cause an avoidance reaction without overt pain [6]. Although there is no consensus definition of photophobia in the field, this definition proposed by Digre and Brennan seems more encompassing and descriptive.

## Etiologic classification

The etiology of photophobia can be subdivided in four main sections: (1) Orbital and visual pathway pathology (e.g., ocular disorders, optic nerve and chiasm problems); (2) Neurological disorders (e.g., primary headache, blepharospasm, traumatic brain injury), (3) Psychiatric disorders (e.g., agoraphobia, anxiety disorder, depression.); and (4) Drug-induced photophobia (e.g., barbiturates, benzodiazepines, haloperidol.)

A number of neurologic conditions are associated with photophobia (Table 1). The most common neurological conditions encountered are primary headaches, benign essential blepharospasm (BEB), Progressive supranuclear palsy (PSP) and traumatic brain injury (TBI) [7]. This review will focus on photophobia present in these conditions.

**Table 1:** Neurological conditions associated with photophobia

Neurological Conditions	
Primary headaches (most common in Migraine)	
Secondary headaches	
Blepharospasm	
Progressive supranuclear palsy	
Traumatic brain injury	
Meningitis	
Subarachnoid hemorrhage	
Lesions of the thalamus	

## Photophobia in neurologic disorders

### Primary headaches

Primary headaches include migraine, tension-type headache (TTH), cluster headache and other trigeminal autonomic cephalalgias, and other less frequent headache types according to the International Classification of Headache Disorders, 3rd edition (ICHD-III) [8]. Photophobia is a common and debilitating symptom often present in migraine and other primary headaches. In a population-based epidemiological study of primary headache in Croatia in 2014 (*n* = 2350), up to 40% of patients reported the symptom [9].

Photophobia is noted in 80%–90% of patients with migraine, which is higher than that in other primary headaches [10, 11]. Patients with migraine experience the symptom both during and between the attacks [12], but it occurs more during the attack than after [13]. Recent research has shown a clear association between migraine-related allodynia and photophobia only in chronic migraineurs [14, 15]. These findings suggest that light stimulation may contribute to central sensitization of pain pathways in migraineurs, possibly contributing to progression into chronic forms. Although the exact prevalence of photophobia in TTH and cluster headache patients is not precisely known, clinical studies showed that subjects with TTH and with cluster headaches had more light sensitivity than controls [16, 17]. One study showed that patients with TTH had lower discomfort thresholds to white light than controls, but had higher thresholds than patients with migraine. It may explain why photophobia is more common in patients with migraine than in patients with TTH [18], and indicates that light sensitivity may be present, but not noticeable to every patient with TTH. Whether the symptom can be unilateral or not still remains controversial. Experimental evidence showed that photophobia is bilateral even when the headache is unilateral in primary headaches [13, 17]. On the other hand, unilateral photophobia has been reported with cluster headache, hemicrania continua, and other trigeminal autonomic cephalagias [19, 20]. A prospective clinical study of short-lasting unilateral neuralgiform headache attacks with conjunctival injection and tearing or cranial autonomic features showed that unilateral photophobia is present in more than 40% of patients [21]. More work is necessary to describe the exact clinical features (bilateral or unilateral) of photophobia by using a more detailed questionnaire.

### Blepharospasm

Blepharospasm, often called benign essential blepharospasm (BEB), is one of the most common focal dystonias. It is characterized by involuntary orbicularis oculi muscle spasms that are usually bilateral, synchronous, and symmetrical [22]. Photophobia is a prominent complaint of patients with blepharospasm. One study demonstrated that patients with blepharospasm were as light sensitive as patients with migraine between the migraine attacks, and that both groups were more light sensitive than controls [23]. In a survey of 316 blepharospasm patients, 94% reported light sensitivity; ambient lighting could provoke spasms about half of the time, but bright light provoked spasms almost all of the time [24]. Another study comprising 240 BEB patients showed that photophobia was present in 25% of patients prior to the onset of the blepharospasm, and up to 74% patients reported the photophobia at the time of the neurological examination [25]. Furthermore, photophobia is considered the second most common factor which can impact BEB patients’ quality of daily life. The mechanism of photophobia is still elusive. A case-control study studied the effect of photochromatic modulation with tinted lenses on the sensory symptoms of photophobia in blepharospasm patients [26]. The results indicated that wavelength of light exposure may influence the symptoms of photophobia in addition to the actual light intensity. In contrast, another study found that the relevant feature for photophobia is the light intensity and not the wavelength at least as altered by the FL-41 lens [23]. Whether symptoms are better relieved by reducing light in a specific region of the spectrum or just reducing the net flux is still controversial.

### Progressive supranuclear palsy

Progressive supranuclear palsy (PSP), previously referred to as Steele Richardson Olszewski syndrome, is a Parkinsonian Syndrome. Characteristic features of PSP include vertical supranuclear gaze palsy and postural instability with falls. A clinical cohort study of 187 patients with PSP showed that photophobia occurred in 43% of patients [27]. Another prospective study showed that photophobia is significantly more frequent in clinically diagnosed PSP than corticobasal degeneration (CBD) (100% vs 18%, *p* = 0.0002) [28]. These results suggest that the presence of photophobia could be used to help clinicians differentiate the two diseases.

### Traumatic brain injury

Traumatic brain injury (TBI) can manifest with visual dysfunction including deficits in accommodation, vergence movements, versions, and field of vision as well increased photosensitivity [29, 30]. Photophobia is one of the most common visual complaints [31]. Studies of TBI have frequently addressed the issue of photosensitivity and its prevalence. One study showed a prevalence of 50% in patients compared with 10% health controls [32]. Photosensitivity does not only occur in the acute phase of brain injury, but also in the chronic phase. However, it is still unclear if photophobia is a primary or secondary symptom of the brain injury. Furthermore, studies revealed that over years, about half of the patients reported reduced photosensitivity (i.e., 10% in the 1st year plus 40% after the 1st year), while 42% remained the same, 3% increased and 5% waxed and waned [33]. The decrease in photosensitivity may be a result of neural repair, neural adaptation and/or compensatory mechanisms.

## Mechanism of photophobia

Since light is the cardinal stimulus for photophobia, photoreceptors must be involved. There are at least five different types of photoreceptors in humans: three kinds of cones, rods and ipRGCs. Rods and cones in the outer retina are the predominant photoreceptor cells of the mammalian retina. Their high temporal and spatial sensitivity to light forms the basis of image-forming vision. The intrinsically photosensitive retinal ganglion cells (ipRGC), which contain the melanopsin photopigment (HUGO gene symbol OPN4), have been identified in recent years [34]. The ipRGC cells are atypical retinal photoreceptors separate from classical rod and cone photoreceptors. Several studies demonstrated that ipRGC play an important role in non-image-forming light effects [35]. The ipRGC cells send their axons to the suprachiasmatic nucleus and the Edinger-Westphal nucleus. In the suprachiasmatic nucleus, these cells entrain circadian rhythms [36]. In the Edinger-Westphal nucleus, they control the pupillary light reflex [34, 36, 37]. Furthermore, evidence accumulating from animal models shows that ipRGCs are involved in photophobia of rod- and cone deficient mice. [38, 39]. In humans, ipRGCs were implicated in photophobia since migraine patients who became blind from a complete lack of rod and cone function still experience photoallodynia compared with patients with enucleated eyes [40]. Melanopsin, the ipRGC photopigment, has peak sensitivity at 480 nm. Using the photic blink reflex, one study tried to objectively quantify the ocular sensitivity to red (640 nm) and blue (485 nm) light in patients with light sensitivity and normal subjects. The data demonstrated an increased photic blink reflex to blue light as opposed to red light in light-sensitive patients [41]. These results suggest that the melanopsin signaling system may control light aversion and the ipRGC may be the most important photoreceptor cell in the pathophysiological mechanism of photophobia.

Although the discovery of the ipRGC cells appear to make an advancement in understanding the mechanism of photophobia, how light functions as a pain stimulus remains elusive. Increasing evidence demonstrates that at least 3 pathways can transmit this signal to the brain. The first pathway was described by Okamoto et al. [42]. They investigated the pattern of neuronal activation in the caudal brainstem after bright light stimulation using quantitative Fos-like immunoreactivity in anesthetized rats. The results showed that light activated the trigeminal brainstem neurons through photoreceptors in the retina (whether rod, cone or ipRGC), which in turn evoked ocular vasodilation and activation of pain-sensing neurons on blood vessels [42]. The second pathway for photophobia is a direct connection between the ipRGC cells and thalamic nuclei, which are associated with somatic sensation and pain [43]. The discovery of this second pathway is particularly significant as the thalamus is an important center for sensory integration, and has important connections to somatosensory centers of the cortex [43]. A third pathway is suggested that does not involve the optic nerve. The ipRGCs and/or ipRGC-like melanopsin-containing neurons in the iris can contribute to the trigeminal afferents bypassing the optic nerve [44, 45].

Neuropeptides that enhance synaptic transmission may play a role in photophobia at multiple levels of the visual and trigeminal pathways. Two particular candidates are the calcitonin gene-related peptide (CGRP) and pituitary adenylate cyclase-activating polypeptide (PACAP), both of which may play a role in migraine attacks in migraineurs [46]. These multifunctional peptides are widely distributed in the nervous system and in addition to their vascular actions; they are both implicated in modulating nociception. Pre-clinical studies have linked both neuropeptides to photophobia. Animal models showed that mice with a gain-of-function mutation of CGRP receptor, exhibit light avoiding behavior when they receive injections of calcium gene-related peptide [47]. Mice lacking PACAP do not develop nitroglycerin-induced light aversion.

## Diagnosis and evaluation of photophobia

Generally, the diagnosis of photophobia is established in the history, along with neurologic and neuro-ophthalmic examination. However, answering the usual question, “does light bother you,” with a “no” should not be taken as indicative of the absence of photophobia [48]. Further questioning with the use of more detailed closed-ended questions is needed to detect the symptom and to evaluate the severity. Assessment tools for photophobia have been sparse. Bossini et al. developed and validated a photophobia questionnaire, a self-assessment tool established in Italian populations [49] (Table 2). It consists of 16 items investigating psychopathological traits and behavioral sensitivity to light. The questions try to identify specifically both behaviors that actively avoid light, termed photophobia (items 2, 6, 7, 9, 10, 12, 13, 14) and that actively search light, described as photophilia (items 1, 3, 4, 5, 8, 11, 15, 16), which have been identified as relevant to the Mediterranean population in clinical practice. For each item the patient may respond in a dichotomous way (yes or no). Affirmative answers are rated as 1 and negative ones as 0, except for item 5 where the scores are reversed (yes = 0, no = 1). Two scores are obtained by the simple sum of each item divided by the number of items for each dimension (8 for photophobia and 8 for photophilia); therefore, two scores ranging from 0 to 1 identify photophobic and photophilic behavior, respectively). Recently, Choi et al. validated a questionnaire in order to assess light aversion behavior in a reproducible way (Table 3) [50]. Their questionnaire seems to be a useful method for detecting photophobia in patients with migraine in Korea. Although this questionnaire has not been validated in populations outside Korea, it may be useful to clinical researchers who are trying to better understand photophobia.

**Table 2:** Photosensitivity Assessment Questionnaire (PAQ)

Photosensitivity Assessment Questionnaire (PAQ)	
1. I prefer summer to winter because winter dreariness makes me sad. (Phi)	
2. If I could, I would be happier to go out after dusk rather than during the day. (Pho)	
3. Often in winter, I’d like to go to the other hemisphere where it is summer time. (Phi)	
4. My ideal house has large windows. (Phi)	
5. I like cloudy days. (Phi)	
6. Sunlight is so annoying to me, that I have to wear sunglasses when I go out. (Pho)	
7. I prefer to stay at home on sunny days, even if it is not warm. (Pho)	
8. I feel reborn in spring when the days start to become longer. (Phi)	
9. Usually strong sunlight annoys me. (Pho)	
10. I prefer rooms that are in semi-darkness. (Pho)	
11. I prefer sunlight to semi-darkness. (Phi)	
12. Looking at a very bright view annoys me. (Pho)	
13. I can’t stand light reflecting off snow. (Pho)	
14. I think summer annoys me because it’s too bright. (Pho)	
15. Sunlight is like therapy for me. (Phi)	
16. I prefer walking in the sunlight if the weather is cool. (Phi)	

Phi: photophilia; Pho: photophobia

These questions try to identify specifically both behaviors that actively avoid light, termed photophobia (items 2, 6, 7, 9, 10, 12, 13, 14) and that actively search light, described as photophilia (items 1, 3, 4, 5, 8, 11, 15, 16), which have been identified as relevant to the Mediterranean population in clinical practice. For each item, the patient may respond in a dichotomous way (yes or no). Affirmative answers are rated as 1 and negative ones as 0, except for item 5 where the scores are reversed (yes = 0, no = 1). Two scores are obtained by the simple sum of each item divided by the number of items for each dimension (8 for photophobia and 8 for photophilia); therefore, two scores ranging from 0 to 1 identify photophobic and photophilic behaviors, respectively

**Table 3:** Photophobia questionnaire for patients with primary headaches (English version of the questionnaire developed by Choi et al. [51])

1	During your headache, do you feel a greater sense of glare or dazzle in your eyes than usual by bright lights?	Yes	No
2	During your headache, do flickering lights, glare, specific colors or high contrast striped patterns bother you or your eyes?	Yes	No
3	During your headache, do you turn off the lights or draw a curtain to avoid bright conditions?	Yes	No
4	During your headache, do you have to wear sunglasses even in normal daylight?	Yes	No
5	During your headache, do bright lights hurt your eyes?	Yes	No
6	Is your headache worsened by bright lights?	Yes	No
7	Is your headache triggered by bright lights?	Yes	No
8	Do you have any of the above symptoms mentioned even during your headache-free interval?	Yes	No

The score ranges from 0 (no photophobia) to 8 (severe photophobia)

## Treatment

Since the mechanism of the photophobia is still not clear, the pharmacotherapeutic treatment remains unknown. There is only little evidence that shows that the systemic medication can relieve the photophobia itself, but treating the underlying conditions may improve the associated photophobia, such as migraine preventive or specific medication (e.g., beta-blockers, calcium channel blockers, anti-convulsants, CGRP-P) for patients with migraine associated photophobia [51, 52, 53].

Botulinum neurotoxin (BoNT) is the treatment of choice for blepharospasm and it also has some efficacy in migraine headache. However, there is limited information for the effect of BoNT on photophobia. A cohort study showed that injection of onabotulinumtoxinA is helpful for photophobia associated with TBI [54]. However, the efficacy needs to be further explored in well-designed studies involving a large population of patients. More work is necessary to evaluate the efficacy of the BoNT in the treatment of photophobia in other conditions as well [54].

Different photoreceptors have different spectral sensitivity to light. Maximum light sensitivities of human rods (R), S cones, M cones and L cones are 500 nm, 420 nm, 530 nm and 560 nm wavelength, respectively. The ipRGCs exhibit their peak sensitivity at 480 nm. There is some evidence that patients with blepharospasm reported the best relief of photophobia with the FL-41 lens, which blocks wavelengths around 480 nm [55]. The use of dark sunglasses indoors must be strongly discouraged. By wearing dark glasses indoors, patients are dark adapting their photorecetors and aggravating their sensitivity to light. These patients should be encouraged to transition to the use of FL-41 or other tints for indoor light sensitivity [7].

## Conclusions

Photophobia is a common symptom seen in many neurologic disorders. While the underlying mechanism of photophobia is still elusive, the discovery of ipRGCs appears to be an advance in understanding. The calcium gene-related peptide receptors (for CGRP and PACAP)) may be potential targets in the treatment of headache and photophobia as well. Generally, the diagnosis of photophobia is established upon the history, along with neurologic and neuro-ophthalmic examination. Since a number of common ophthalmic conditions are associated with photophobia, patients should be referred to an ophthalmologist to rule-out or treat these conditions if the neurologic history and examination fail to suggest a diagnosis. Furthermore, physicians should be encouraged to use an assessment tool of photophobia or photophilia for detecting the symptoms. Wearing specially tinted spectacles may provide an effective means to relieve the photophobia.

## Abbreviations

BEB: Benign essential blepharospasm
BoNT: Botulinum neurotoxin
CBD: Corticobasal degeneration
CGRP: Calcitonin gene-related peptide
CNS: Central nervous system
ipRGC: Intrinsically photosensitive retinal ganglion cells
PACAP: Pituitary adenylate cyclase-activating polypeptide
PSP: Progressive supranuclear palsy
TBI: Traumatic brain injury
TTH: Tension-type headache
